# Cancer of the Larynx

**Published:** 1908-01-18

**Authors:** 


### Laryngology and Rhinology.

### CANCER OF THE LARYNX.

The only form of malignant growth which is found with any frequency within the larynx is epithelioma, and therefore the statements in the following article will apply chiefly to this affection. An epithelioma originating in the neighbourhood of the vocal cords is probably, under certain circumstances, the least unfavourable of all forms of malignant disease, and this for several reasons. A growth in this region tends to spread slowly at first, and infection of the lymphatic glands does not occur early in the disease. Moreover —and this is the most important reason—hoarseness is pronounced at an early stage, while the growth is still very minute, and it can be definitely seen and diagnosed by ocular inspection through the laryngoscope, when no larger than the head of a steel pin, and when, if situated in almost any other part of the body, its presence would not even be suspected. The moral of this is to examine with the laryngoscope every case of hoarseness, at any rate every intractable case, especially if it occur in an elderly patient; though it must also be remembered that epithelioma of the larynx is sometimes found at a comparatively early age, and is not rare after the thirtieth year.

### Symptoms.

The only early symptom at all common is hoarseness. The reason of the rapid and marked onset of this symptom is probably to be found in the early infiltration of the underlying muscles. It appears most quickly when the growth is situated on the vocal cord, but it also occurs fairly soon in cancer of other parts of the larynx, as a result of fixation of the arytenoid, or of infiltration and stiffness of the tissues. The voice has neither the rough and raucous tone of syphilis, nor the weak aphonic quality of tuberculous laryngitis; it is usually gruff, and may be sufficiently characteristic to arouse suspicion, though too much stress must not be placed upon this point.

Pain, especially on swallowing, and obstructive dysphagia, occur early in cases of cancer situated outside the lumen of the larynx, about the upper aperture, or on the back of the cricoid plate; but there is rarely any pain in the early stages of growths originating within the cavity of the organ, this symptom only appearing when the disease has spread beyond the lumen. The pain is sharp, and frequently shoots upwards to the ears; it occurs spontaneously, but is made worse by speaking and coughing, and especially by swallowing.

Respiration may be obstructed by the mass of the growth, but sometimes also by fixation of the arytenoids or by abductor paralysis, the result of pressure on the recurrent nerves, or of involvement of the abductor muscles on the back of the cricoid plate.

Cough is not a marked symptom. Salivation is frequently present in the later stages; fœtor is then common, and pneumonia from inhalation of septic particles is a frequent cause of death in these cases. Failure of health, loss of appetite, and general cachexia usually appear early. A small amount of bleeding often occurs when the disease is far advanced, but occasionally severe hæmorrhage takes place at quite an early stage.

### Objective Appearances and Diagnosis.

Krishaber has divided cases of laryngeal cancer into two classes, *instrinsic* and *extrinsic*; and this classification is of practical value, and is generally followed.

Intrinsic growths are those which originate on the vocal cords, ventricular bands, ventricles, inter-arytenoid space, and the subglottic region—all those, in fact, which begin within the lumen of the larynx.

Extrinsic cancer attacks the upper aperture and outer surface of the larynx, such as the posterior surface of the cricoid plate. This latter situation is affected with especial frequency in women; but it is not strictly a laryngeal cancer, as it originates on the mucous membrane of the pharynx. In the later stages a growth will often be both intrinsic and extrinsic. Intrinsic tumours grow, as a rule, more slowly, and secondary infection of the lymphatic glands does not readily occur; extrinsic cancers, on the contrary, extend rapidly, infect the lymphatics at an early stage, soon cause pain and dysphagia, and demand a much more formidable operation for their extirpation.

### Epithelioma on the Vocal Cords.

On the vocal cord epithelioma may, at its first appearance, assume a great variety of forms, but three principal types may be distinguished:—(1) A single polypoid outgrowth; (2) a diffuse infiltration; or (3) a unilateral congestion. In the first form the tumour may present a smooth or a warty surface, and may be white, grey, or red. It is sessile, and from the first it grows *into* the substance of the cord, and thus gives rise to the most characteristic signs. Thus there is a delayed, sluggish movement of the cord, which, as pointed out by Semon, is one of the most important signs of malignancy; of course it is not always to be found, and impairment of mobility occurs in other infections, but it is sufficiently common, and usually well enough marked, to be of great diagnostic value.

An innocent tumour is generally pedunculated; it may overlie its pedicle, but it is mobile on phonation, and, on touching it with a probe or forceps, it moves freely, and pulls with it a fold of mucous membrane, whereas this does not occur in the case of an epithelioma. In the latter, too, there is often some tumefaction beyond the obvious limits of the growth, and enlarged vessels, coursing over the surface of the cord to the tumour, are strongly suggestive of malignant disease. An innocent tumour generally grows from the centre of the true cord, that is, at the junction of the anterior and middle thirds of the entire length of the glottis; a tumour originating at any other part of the cord is more likely to be malignant.

(*To be continued.*)

